# Serotonin deficiency induced after brain maturation rescues consequences of early life adversity

**DOI:** 10.1038/s41598-021-83592-4

**Published:** 2021-03-08

**Authors:** B. Aboagye, T. Weber, H. L. Merdian, D. Bartsch, K. P. Lesch, J. Waider

**Affiliations:** 1 Division of Molecular Psychiatry, Laboratory of Translational Neuroscience, Center of Mental Health, University of Würzburg, Margarete-Höppel-Platz 1, 97080 Würzburg, Germany; 2 Laboratory of Psychiatric Neurobiology, Institute of Molecular Medicine, I.M. Sechenov First Moscow State Medical University, Moscow, Russia; 3 Department of Psychiatry and Psychology, School for Mental Health and Neuroscience (MHeNS), Maastricht University, Maastricht, The Netherlands; 4 Department of Molecular Biology, Central Institute of Mental Health, Medical Faculty Mannheim , Heidelberg University, Mannheim, Germany; 5 MEDIAN Clinic Wilhelmsheim, Oppenweiler, Germany; 6 School of Psychology, University of Lincoln, Lincoln, UK; 7 Department of Biomedical and Forensic Science, School of Biological Sciences, University of Cape Coast, Cape Coast, Ghana

**Keywords:** Neuroscience, Emotion, Molecular medicine

## Abstract

Brain serotonin (5-HT) system dysfunction is implicated in depressive disorders and acute depletion of 5-HT precursor tryptophan has frequently been used to model the influence of 5-HT deficiency on emotion regulation. Tamoxifen (TAM)-induced Cre/loxP-mediated inactivation of the tryptophan hydroxylase-2 gene (*Tph2*) was used to investigate the effects of provoked 5-HT deficiency in adult mice (*Tph2* icKO) previously subjected to maternal separation (MS). The efficiency of Tph2 inactivation was validated by immunohistochemistry and HPLC. The impact of *Tph2* icKO in interaction with MS stress (*Tph2* icKO × MS) on physiological parameters, emotional behavior and expression of 5-HT system-related marker genes were assessed. *Tph2* icKO mice displayed a significant reduction in 5-HT immunoreactive cells and 5-HT concentrations in the rostral raphe region within four weeks following TAM treatment. *Tph2* icKO and MS differentially affected food and water intake, locomotor activity as well as panic-like escape behavior. *Tph2* icKO prevented the adverse effects of MS stress and altered the expression of the genes previously linked to stress and emotionality. In conclusion, an experimental model was established to study the behavioral and neurobiological consequences of 5-HT deficiency in adulthood in interaction with early-life adversity potentially affecting brain development and the pathogenesis of depressive disorders.

## Introduction

Brain serotonin (5-HT) system dysfunction is implicated in disorders of emotion regulation, such as anxiety and depression, viewed as multifactorial conditions influenced by multiple gene-by-gene and gene-by-environment interactions^1^. Previous research identified genetic variants regulating the expression of the gene encoding the rate-limiting enzyme of neuronal serotonin (5-HT) synthesis, tryptophan hydroxylase-2 (TPH2)^2,3^, which converts the essential amino acid tryptophan (TRP) into 5-OH-TRP, the direct precursor of 5-HT^4,5^.

Depletion of TRP by dietary intervention (acute TRP depletion, ATD) has traditionally been used in assessing the influence of 5-HT deficiency on emotion dysregulation and the pathogenesis of depressive disorders. TRP-free diet produces an acute and profound reduction in brain TRP and consequently brain 5-HT synthesis^6–8^. ATD decreases anxiety in patients with anorexia nervosa^9^ and also lowered mood in healthy participants and individuals with bulimia nervosa^10,11^. However, the mechanisms of ATD and the impact of reduced brain 5-HT on behavior are still not well understood^12^. Animal model studies provide evidence for alterations in brain 5-HT release following ATD, resulting in altered anxiety- and depression-related behavior^13^.

Genetic manipulation in mice, which results in embryonic inactivation of *Tph2* or other 5-HT neuron development-related genes (e.g. *Pet1*, *Lmx1b*), provided insight into the behavioral and physiological consequences of 5-HT deficiency in the brain^14,15^. Several studies showed that neonates with a constitutive *Tph2* inactivation present viability and growth-related problems compared to wildtype counterparts^16,17^. While these mice retain normal 5-HT neuron morphology and physiology, and are indistinguishable from controls in adulthood^18,19^, several behavioral phenotypes have been reported^20–22^. These phenotypes arising from lifelong *Tph2* inactivation may be the consequence of impaired functioning of other relevant genes, the expression of which is, at least partly, dependent on 5-HT during brain maturation^23,24^ and may result in structural and functional differences of the networks involved in emotional processing of mice lacking Tph2^25^.

Here, we used Tamoxifen (TAM)-induced Cre/loxP-mediated inactivation of the *Tph2* to investigate the effects of provoked 5-HT deficiency in the modulation of emotional responses and risk for anxiety disorders and depression in interaction with environmental adversity during brain maturation in adult mice (*Tph2* icKO) previously subjected to maternal separation (MS).

## Material and methods

### Animals

In this study only male mice were used. They were housed in groups in a controlled environment (12/12 h light/dark cycle, 21 ± 0.5 °C room temperature, 50 ± 5% humidity) with food and water ad libitum. Mice were acclimatized to single housing conditions for ≥ 3 weeks prior to behavioral experiments. Behavioral tests were performed during the light phase between 10:00 and 15:00 with a recovery period of 7 days between different tests. All in vivo animal experiments were performed in accordance with the European Parliament and Council Directive (2010/63/EU) and ARRIVE guidelines. The study was approved by the institutional review board of the University of Würzburg and the Government of Lower Franconia (55.2-2531.01-57/12).

### Induction of Tph2 inactivation

For temporal and spatial control of *Tph2* recombination, mice homozygous for *Tph2* exon five flanked by loxP-sites (*Tph2*^fl/fl^), the palindromic recognition sites of Cre recombinase^26^ backcrossed onto a C57Bl/6N background were crossed with Tph2 null mutant mice (*Tph2*^−/−^)to generate *Tph2*^*fl*/−^ hemizygous mice, which lack one *Tph2* allele from the beginning of their life similar to heterozygous *Tph2* knockout mice (*Tph2*^+/−^). These mouse lines were crossed with C57Bl/6N.Tg^Tph2creERT2^ transgenic mice, which express CreERT2 under the control of the murine Tph2 promoter exclusively in the raphe nuclei , to generate Tg^Tph2creERT2^/*Tph2*^*fl*/*fl*^ and Tg^Tph2creERT2^/*Tph2*^*fl*/−^ mice respectively. Tg^Tph2creERT2^/*Tph2*^*fl*/*fl*^ or Tg^Tph2creERT2^/*Tph2*^*fl*/−^ males were crossed with *non-transgenic* females in order to control the number of Tph2creERT2 transgenes (*n* = 1) in the animals. In order to initiate Cre/loxP-mediated recombination to generate an induced raphe nuclei specific Tph2 knockout (*Tph2icKO*), male Tg^Tph2creERT2^/*Tph2*^*fl*/*fl*^ or Tg^Tph2creERT2^/*Tph2*^*fl*/−^ mice aged 10–12 weeks were injected twice a day for 5 consecutive days with 1 mg of Tamoxifen (TAM; Sigma Aldrich, St. Louis, USA)^27^ resulting in *Tph2*^Δfl/−^ mice or *Tph2*^Δfl/fl^. In this respect*Tph2*^Δfl/−^ mice model an *Tph2icKO* based on a hemizygous background, while *Tph2*^Δfl/fl^ mice model an *Tph2icKO* based on a wildtype like genetic background during development. Tg^Tph2creERT^/*Tph2*^+/+^ mice injected with TAM (*Tph2*^Δ+/+^) were used as controls. Vehicle injected mice of same genotype were used as controls in the maternal separation experiments (*Tph2* CON)*.*

### Immunohistochemistry

The efficacy of TAM-induced time-specific *Tph2* inactivation in the brainstem raphe region was assessed by fluorescence immunohistochemistry in the 4th and 6th week after treatment. Thin sections (30 µm) of brain of mice (n = 7–9/group) were cut on cryostat and stored at − 80 °C. Frozen sections were dried for 15–20 min. An antigen retrieval was conducted as previously described^26^. Sections were cooled down to 40 °C and washed 3 × 5 min in Tris-buffered saline (TBS). Unspecific binding sites for the antibodies were blocked for 90 min at room temperature (RT) with blocking solution (5% normal goat serum, 0.25% Triton-X100 in TBS). Sections were incubated overnight with the primary antibody (1:400, goat-anti 5-HT; Immunostar; Hudson; USA) diluted in blocking solution (TBS-T) at 4 °C in a humid chamber. Following three 5-min washing steps in TBS, sections were incubated in the dark with the respective fluorescent secondary antibodies (1:400 Cy3) diluted in blocking solution, lasting 90 min. Sections were washed 3 × 5 min in TBS. For staining of the cell nuclei, sections were treated with 300 µm DAPI diluted 1:1000 in TBS for 5 min. The number raphe neurons that were immunoreactive (ir) for 5-HT in dorsal (DRN: B6, B7) and median raphe nuclei (MRN: B5, B8, B9)^28,29^ based on cell nuclei surrounded by anti-Tph2 signal using Fiji software^30^ on four consecutive pictures of anterior raphe (DRN and MRN) corresponding to Bregma—4.95 mm to—4.47 mm spaced 180 µm^31^.

### Neurochemistry

For high performance liquid chromatography (HPLC), three brain regions (hippocampus, the dorsal raphe and the amygdaloid complex) were quickly dissected under a stereo microscope. For this, the brain (n = 4–7) was sliced with the aid of a metallic matrix, which allows sectioning at equal intervals on a cold plate. The identified regions were dissected out with a preparation spatula and kept frozen at − 80 °C until use. The brain homogenates were prepared and analyzed to standard protocols^19,25^.

### Body weight and food intake

Body weight of mice (n = 18–25) used for the baseline behavioral study was measured weekly for 7 weeks starting from the first week of injection. Body-weight measurements were conducted from 10 to 11 a.m. Two weeks after the last injection, mice were single-housed and their food and water-intake was measured weekly.

### Behavioral assessment

Mice were subjected to behavioral testing starting from 4 weeks after TAM injections. In the baseline study, one group of mice (*n* = 13–15/group) was first tested for anxiety-like behavior in the light–dark transition test (LDT), followed by an open-field test (OF) to assess locomotor activity in a novel inescapable environment^32^. A second group of mice (*n* = 7–11/group) was used to assess anxiety- and depression-like behaviour including an elevated-plus maze test (EPM), sucrose preference test and lastly Porsolt swim test (PST)^33^. Observations were recorded with VideoMot2 (TSE Systems, Bad Homburg, Germany) and later analyzed with EthoVision XT 11.5 (Noldus, Wageningen, The Netherlands). For details see supplementary methods.

### Maternal separation

In the second study, pups of the Tg^Tph2creERT2^/*Tph2*^fl/fl^ genotype were separated on the second postnatal day (P2) from their mothers and kept in fresh cages for 3 h daily (between 10.00 and 13.00), for 14 consecutive days (P2-16). Ambient temperature was ensured by infrared light, positioned 70 cm above the cage. Non-MS mice were not separated from the dams but were handled during routine cage changes. Mice were weaned at 25 days after birth and kept in groups of 2 to 5 mice per cage and injected with either TAM (*Tph2*^*Δfl*/*fl*^) or vehicle (CON) (n = 8–10/group). After 4 weeks, mice were tested for anxiety-related behavior in the EPM, LBD and OF followed by SPT and FST for depression related behavior with 3 days inter-trial time, all tests were done as described in supplementary methods.

### Quantitative real-time PCR

Quantification of relative gene expression was performed by quantitative real-time PCR (qRT-PCR). cDNA was generated as previously described^34^. The reaction was run in triplicates using SYBR green dye according to manufacturer instructions. Reaction mixture comprised 6 µl (SYBR green + Primer (F + R)) mix and 4 µl cDNA making 10 µl reaction volume each. Mean efficiencies were calculated by LinReg^35^. Relative expression data were calculated by qBase + (Biogazelle, Zwijnaarde, Belgium), with the normalization factors obtained from geNorm (geNorm M < 0.5)^36^. Reference genes: *glyceraldehyde 3-phosphate dehydrogenase (GAPDH_3)*, *beta-2 microglobulin (B2m_2)*, *ubiquitin C (UBC_1) and ribosomal protein lateral stack subunit P0 (Rplpo)*. Selected target genes were *tryptophan hydroxylase 2 (Tph2)*, *5-HT receptor 1a (Htr1a)*, *5-HT receptor 2a (Htr2a)*, *monoamine oxidase A (Maoa)*, *arginine vasopressin receptor 1a (Avpr1a)*.

### Data analysis

Data obtained from this study were analyzed and displayed using GraphPad Prism version 6.07 for Windows (GraphPad Software, La Jolla California USA, www.graphpad.com). The total numbers of 5-HT ir cells counted in the anterior raphe of *Tph2* icKO were compared with that of *Tph2* CON mice using Kruskal–Wallis statistic (*H*), while Dunn’s multiple comparison test was used to compare means between the groups of mice.

Behavioral outcomes on baseline anxiety- and depression-like behavior were analyzed by one-way ANOVA. The course of 5-HT depletion from week 2, 4 and 6 were analyzed by two-way ANOVA as well as MS and gene expression data, with Tukey’s multiple comparison post hoc test used to compare means. Students t-test was used for comparisons between *Tph2*^*Δ*+/+^ and *Tph2*^*fl*/*fl*^ mice (Figs. S1 and S2). An α < 0.05 was set as level of significance.

## Results

### Efficiency of induced Tph2 inactivation

**Figure 1:**
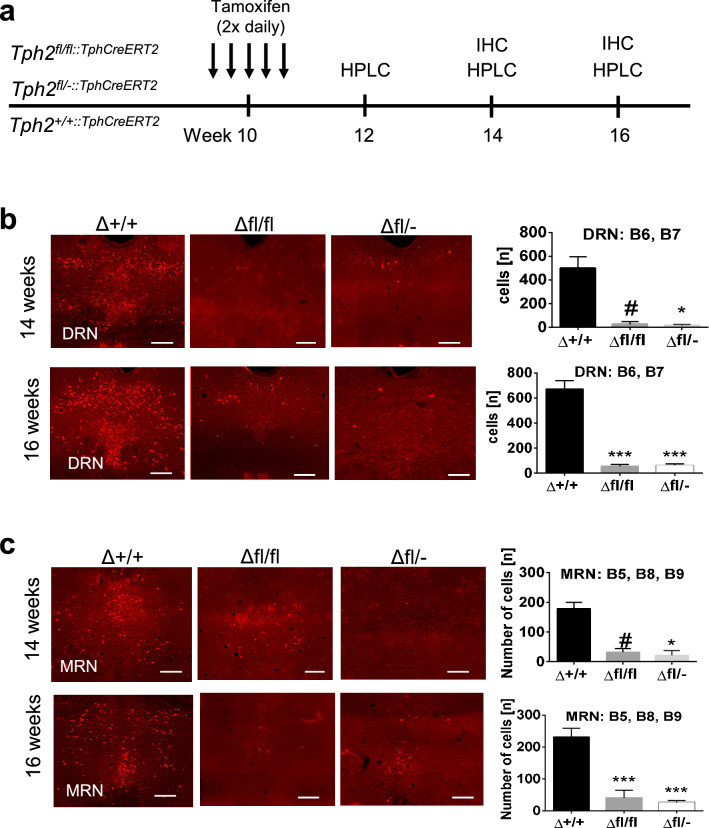
Tamoxifen (TAM)-induced Cre/fl-mediated inactivation of the tryptophan hydroxylase-2 (*Tph2*) gene during adulthood in raphe 5-HT neurons of Tg^Tph2creERT2^/*Tph2*^+/+^ (Δ+/+), Tg^Tph2creERT2^/*Tph2*^fl/fl^ (Δfl/fl) and Tg^Tph2creERT2^/*Tph2*^fl/-^ (Δfl/−) mice. (**a**) Timeline for gene targeting, immunohistochemistry (IHC) and HPLC; (**b**) IHC and quantification of 5-HT positive cells in the dorsal raphe nucleus (DRN) B6 and B7 at week 14 (upper panel) and week 16 (lower panel) after treatment; (**c**) IHC and quantification of 5-HT positive cells in MRN at week 14 (upper panel) and week 16 (lower panel) after treatment. Number of 5-HT positive cells counted represented as mean ± SEM in week 14 (n = 4/group) and week 16 (n = 6–9). Data are shown as mean ± SEM. #0.05 < *p* < 0.1, *p < 0.05 and ***p < 0.001.

The half-life of Tph2 is approximately 2.5 days^37^. Thus, we first evaluated the time required after induced Tph2 recombination to reduce the amount of Tph2 synthesis to a level similar to constitutive *Tph2* KO mice  by immunohistochemistry (Fig. 1a). Four weeks after TAM treatment, effective recombination of *Tph2* was indicated by a significant drop of 5-HT ir cells in the DRN (Fig. 1b) of *Tph2* icKO compared with *Tph2* CON mice [(*H*_2_) = 7.731; *p* = 0.0066; Fig. 1b, right panel]. The number of 5-HT ir cells in the raphe region of *Tph2*^*Δfl*/−^ (*p* = 0.0107) and *Tph2*^Δ*fl*/*fl*^ (*p* = 0.0789) mice was reduced by 97.6% and 95.0% respectively compared to *Tph2*^*Δ*+/+^ mice. After six weeks following TAM injection, only a few scattered 5-HT ir cells were observed in the DRN (Fig. 1b) in *Tph2*^*Δfl*/−^ and *Tph2*^Δ*fl*/*fl*^ in comparison to *Tph2*^*Δ*+/+^ mice (*H*_2_) = 16.43; *p* = 0.0003; Fig. 1b, right panel). *Tph2*^Δ*fl*/*fl*^ (*p* = 0.0006) and *Tph2*^*Δfl*/−^ (*p* = 0.0023) showed a 93.7% and 92.2% reduction of 5-HT ir cells compared to *Tph2*^*Δ*+/+^ mice. Similar to the DRN, the number of 5-HT ir cells in the MRN was decreased in *Tph2* icKO after four weeks ((*H*_2_) = 16.65; *p* = 0.0002) and six weeks after treatment ((*H*_2_) = 7.758; *p* = 0.0062). Compared to *Tph2*^*Δ*+/+^ mice, a significantly reduced number of 5-HT ir cells in *Tph2*^Δ*fl*/*fl*^ (*p* = 0.0053) and *Tph2*^*Δfl*/−^ (*p* = 0.0003) was detected by week four. Similar observations were recorded by week six in *Tph2*^Δ*fl*/*fl*^ (*p* = 0.0783) and *Tph2*^*Δfl*/−^ (*p* = 0.016), which accounted for 92.7% and 82.1% reduction, respectively (Fig. 1c). Of note the number of 5-HT ir cells in DRN and MNR of *Tph2*^*Δ*+/+^ mice did not differ to vehicle treated *Tph2*^*fl*/*fl*^ mice (Fig. S1).

### Neurochemistry

**Figure 2:**
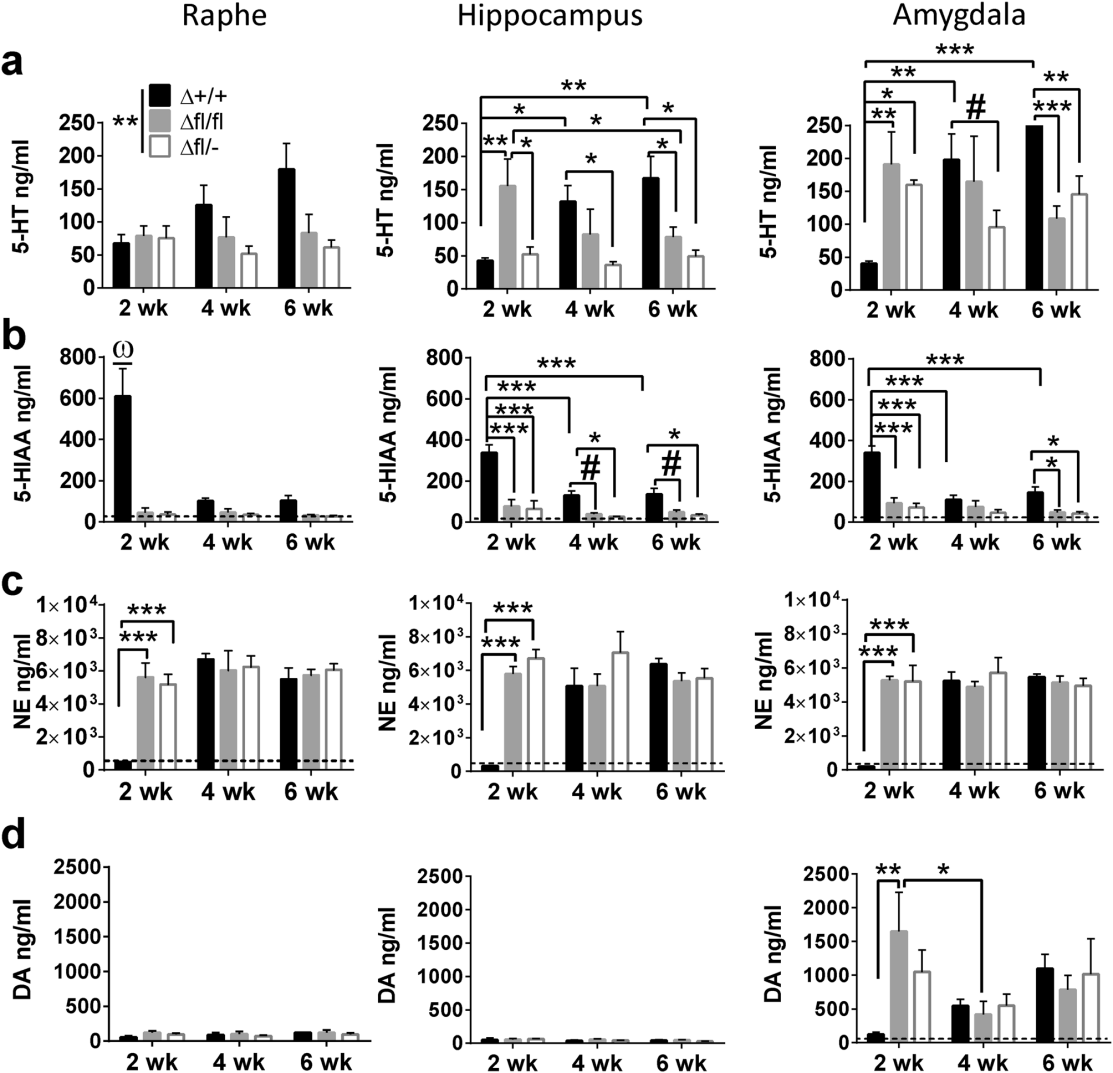
Concentrations of monoamines in brain of *Tph2* induced conditional knockout mice (*Tph2*^+/+^, *Tph2*^*fl*/*fl*^, *Tph2*^*fl*/−^). HPLC analysis of 5-HT in raphe, hippocampus and amygdala at week (wk) 2, 4 and 6 after induction; (**a**) 5-HT, (**b**) 5-HIAA, (**c**) NE, (**d**) DA in selected brain regions. Data are shown as mean ± SEM. ^#^0.05 < *p* < 0.1, **p < 0.01, ***p < 0.001 and ^ω^*p* < 0.001 compared to all groups.

In order to relate the absence of 5-HT ir cells to concentrations of monoamines in the raphe and target brain regions, we measured the concentrations of 5-HT and its metabolite, 5-hydroxyindolacetic acid (5-HIAA), as well as norepinephrine (NE) and dopamine (DA) in the raphe, hippocampus and amygdala at all three time points after TAM induction. Two-way ANOVA revealed a tendency towards significance in the raphe for genotype × time interaction (F_(4,40)_ = 2.299; *p* = 0.0756). Indeed, from week 2 to 6 *Tph2*^*Δ*+/+^ presented increased 5-HT concentrations (*p* = 0.0032) in the brainstem at a comparable level to wildtype (*Tph2*^+/+^) controls^19^, and showed significantly higher concentrations compared to *Tph2*^*Δfl*/−^ (*p* = 0.016) and *Tph2*^Δ*fl*/*fl*^ (*p* = 0.0783) mice. The concentration of 5-HT in *Tph2*^*Δfl*/−^ and *Tph2*^Δ*fl*/*fl*^ mice was relatively stable at all time points, remaining at low concentrations (67.0 ± 13.6 ng/ml), similar to those reported in constitutive *Tph2*^−/−^ mice^19^ (Fig. 2a, left panel). Moreover, the concentrations of the 5-HT metabolite 5-HIAA remained consistently low in both genotypes of *Tph2* icKO mice at all time points examined. However, two-way ANOVA revealed a significant genotype × time interaction (F_(4,40)_ = 8.157; *p* < 0.0001; Fig. 2b, left panel), showing higher 5-HIAA in *Tph2*^Δ+*l*+^ compared to *Tph2*^Δ*fl*/*fl*^ and *Tph2*^*Δfl*/−^ (both *p* < 0.0001) only two weeks after injections.

In the hippocampus a significant genotype × time interaction was detected (F_(4,40)_ = 6.493; *p* = 0.0004; Fig. 2a, middle panel). Similar to the raphe, a rise in 5-HT concentrations in *Tph2*^*Δ*+/+^ mice from week 2 to 6 was observed. The amount of 5-HT detected in *Tph2*^Δ+*l*+^ was considerably higher than *Tph2*^Δ*fl*/*fl*^ (*p* = 0.017) and *Tph2*^*Δfl*/−^ (*p* = 0.0013) in week 6. However, in contrast to the raphe, *Tph2*^Δ*fl*/*fl*^ had higher concentrations of 5-HT at week 2, which declined until week 6, whereas in *Tph2*^*Δfl*/−^ mice low 5-HT concentrations were detected at all time points. This provides evidence that recombination of two functional *Tph2* alleles requires more time to effect changes in 5-HT concentrations in hippocampal projections. Furthermore, hippocampal 5-HIAA concentrations revealed a significant genotype × time interaction (F_(4,40)_ = 4.776; *p* = 0.003; Fig. 2b, middle panel). In week 2, *Tph2*^Δ+*l*+^ displayed significantly higher concentrations of 5-HIAA than *Tph2*^Δ*fl*/*fl*^ and *Tph2*^*Δfl*/−^ (both *p* < 0.0001) like in the brainstem, which was also detected in week 4 [*Tph2*^Δ*fl*/*fl*^ (*p* = 0.0767); *Tph2*^*Δfl*/−^ (*p* = 0.0258)] and week 6 [*Tph2*^Δ*fl*/*fl*^ (*p* = 0.0683); *Tph2*^*Δfl*/−^ (*p* = 0.0285)] also less pronounced.

In the amygdala a significant genotype × time interaction effect was observed (F_(4,40)_ = 9.316; *p* < 0.0001; Fig. 2a, right panel). Similar to the other brain regions, 5-HT concentrations in *Tph2*^*Δ*+/+^ was significantly lower compared to *Tph2*^Δ*fl*/*fl*^ (*p* = 0.0023) and *Tph2*^*Δfl*/−^ (*p* = 0.0214) at week two after induction and increased towards week 6 resulting in higher concentrations of 5-HT than *Tph2*^Δ*fl*/*fl*^ (*p* = 0.0004) and *Tph2*^*Δfl*/−^ (*p* = 0.0044). However, no significant changes were detected over time in *Tph2* icKO mice, the concentration of 5-HT remained constantly high. Yet, the concentrations of 5-HIAA documented in the raphe, amygdala and hippocampus showed a significant genotype × time interaction effect (F_(4,40)_ = 7.356; *p* = 0.0002; Fig. 2b right panel). In week 2, *Tph2*^*Δ*+/+^ displayed significantly higher concentrations of 5-HIAA compared to *Tph2*^Δ*fl*/*fl*^ and *Tph2*^*Δfl*/−^ (all *p* < 0.0001). Whereas in week 4, no significant differences were observed, *Tph2*^*Δ*+/+^ showed higher concentrations of 5-HIAA than *Tph2*^Δ*fl*/*fl*^ (*p* = 0.0184) and *Tph2*^*Δfl*/−^ (*p* = 0.0114) in week 6.

For NE two-way ANOVA revealed significant genotype × time interaction effects in the raphe (F_(4,40)_ = 6.327; *p* < 0.0001; Fig. 2c, left panel), hippocampus (F_(4,40)_ = 9.862; *p* < 0.0001; Fig. 2c middle panel) and amygdala (F_(4,40)_ = 62.804; *p* < 0.0001; Fig. 2c, right panel). *Post-hoc* analyses revealed that in all brain regions *Tph2*^*Δ*+/+^ had lower NE concentrations compared to *Tph2*^Δ*fl*/*fl*^ and *Tph2*^*Δfl*/−^ mice at week 2 (*p* < 0.0001).

Only in the amygdala a significant genotype × time interaction was detected for the concentrations of DA (F_(4,40)_ = 2.822; *p* = 0.038; Fig. 2d, right panel). *Post-hoc* testing showed lower DA in *Tph2*^*Δ*+/+^ compared with *Tph2*^Δ*fl*/*fl*^ (*p* = 0.0024) and *Tph2*^*Δfl*/−^ (*p* = 0.0837) at week 2. At week 4 DA concentrations in *Tph2*^Δ*fl*/*fl*^ dropped significantly compared to week 2 (*p* = 0.0385). At week 6, no differences between genotypes were detected. Increased DA concentrations in*Tph2*^*Δ*+/+^ at week 6 approached significance compared to week 2 (*p* = 0.065), indicating a balancing effect in *Tph2*^*Δ*+/+^ over time. In the raphe (Fig. 2d, left panel) and hippocampus (Fig. 2d, middle panel) DA concentrations remained constant beyond all groups.

### Locomotor hyperactivity but unaltered anxiety- and depression-like behavior in Tph2 icKO mice

**Figure 3:**
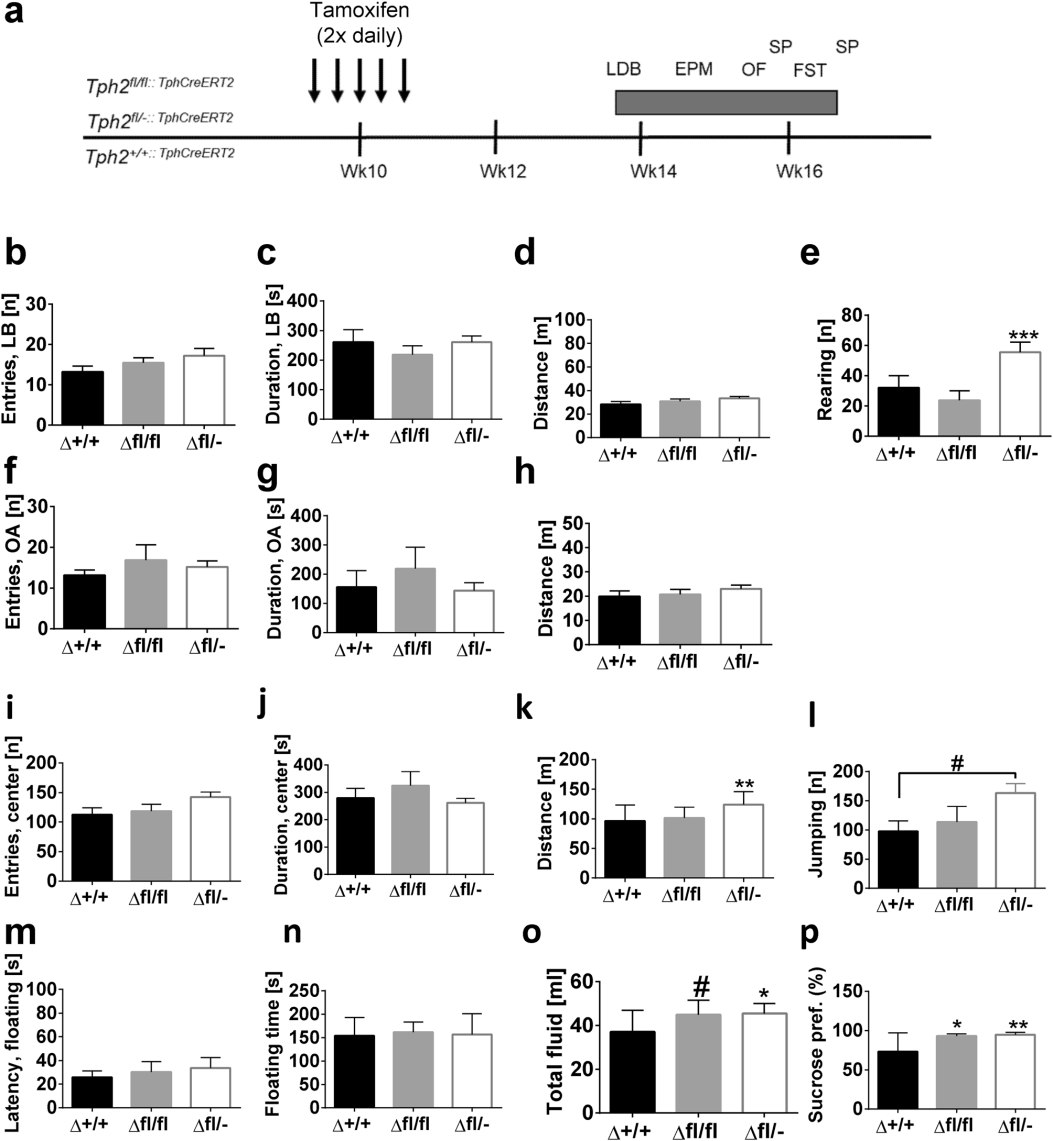
Lack of effects on anxiety- and depression-like but increased exploratory behavior in Tph2 icKO mice based on hemizygous genetic predisposition (**a**) Timelines for behaviour testing; (**b**)–(**e**) LDB: light box visits, time in light box, total distance and vertical rearings; (**f**)–(**h**) EPM: open arms visits, time in open arms and total distance; (**i**)–(**l**) OFT: center visits, time in center, total distance and jumping behavior; (**m**),(**n**) FST: latency to float and floating duration; (**o**),(**p**) SPT: total fluid consumed and sucrose preference. Data are shown as mean ± SEM. #0.05 < *p* < 0.1, **p < 0.01 and ***p < 0.001.

After demonstrating the time course of 5-HT reduction after *Tph2* icKO induction, we investigated anxiety and depressive-like behavior 4–6 weeks after the injections (Fig. 3a). In the OF test, *Tph2*^*Δ*+*/*+^, *Tph2*^*Δfl*/−^ and *Tph2*^Δ*fl*/*fl*^ mice put up similar performances in the frequency of visits to the aversive center (F_(2,39)_ = 2.431, *p* = 0.1012; Fig. 3i) and time spent in center (F_(2,39)_ = 0.8357, *p* = 0.4412; Fig. 3j). *Tph2*^*Δ*+*/*+^, *Tph2*^*Δfl*/−^ and *Tph2*^Δ*fl*/*fl*^ mice differed significantly in the total distance traveled (one-way ANOVA, F_(2,39)_ = 6.224, *p* = 0.0045; Fig. 3k). Indeed, *Tph2*^*Δfl*/−^ traveled longer distances than *Tph2*^*Δ*+*l*+^ and *Tph2*^Δ*fl*/*fl*^ (*p* = 0.004). Furthermore, a tendency in favor of *Tph2*^*Δfl*/−^ was seen in jumping activity (F_(2,39)_ = 2.944, *p* = 0.0644; Fig. 3l). In the LDT, *Tph2*^*Δfl*/−^ and *Tph2*^Δ*fl*/*fl*^ mice did not differ from *Tph2*^*Δ*+*/*+^ mice in all behavioral measures including entries and time spent in the lit compartment as well as total distance traveled (Fig. 3b–d). However, vertical rearing activities varied between groups (F_(2,41)_ = 5.865, *p* = 0.0058). Here *Tph2*^*Δ*+*/*+^ and *Tph2*^*Δfl*/−^ mice differed significantly (*p* = 0.0379; Fig. 3e). In the EPM, *Tph2*^*Δ*+*/*+^, *Tph2*^*Δfl*/−^ and *Tph2*^Δ*fl*/*fl*^ mice showed similar results regarding the frequency of visits to the open arms (F_(2,24)_ = 0.7034, *p* = 0.5048; Fig. 3f), time spent on open arms (F_(2,24)_ = 0.5964, *p* = 0.5587; Fig. 3g) and total distance traveled (F_(2,24)_ = 0.7257, *p* = 0.4943; Fig. 3h).

Behavioral despair and anhedonia were tested in the FST and sucrose preference test. *Tph2*^*Δ*+*/*+^, *Tph2*^*Δfl*/−^ and *Tph2*^Δ*fl*/*fl*^ mice did not show any significant variation in latency to floating (F_(2,24)_ = 0.2681, *p* = 0.7671; Fig. 3m) and duration of immobility (one-way ANOVA, F_(2,24)_ = 0.0737, *p* = 0.9291; Fig. 3n). Of note, *Tph2*^*Δ*+/+^ mice did not differ in behaviour to vehicle treated *Tph2*^*fl*/*fl*^ mice (Fig. S2). *Tph2*^*Δfl*/−^ and *Tph2*^Δ*fl*/*fl*^ mice consumed more fluid than *Tph2*^*Δ*+/+^ mice (F_(2,22)_ = 3.585, *p* = 0.0449; Fig. 3o) with *Tph2*^*Δ*+/+^ consuming significantly less fluid that *Tph2*^*Δfl*/−^ (*p* = 0.0384) and a tendency in comparison with *Tph2*^Δ*fl*/*fl*^ mice (*p* = 0.0992). In the SPT, differences in preference for sucrose were detected (F_(2,22)_ = 5.972, *p* = 0.0085; Fig. 3p) such that *Tph2*^Δ*fl*/*fl*^ (*p* = 0.0301) and *Tph2*^*Δfl*/−^ (*p* = 0.0076) preferred sucrose more than *Tph2*^*Δ*+/+^.

Finally, we assessed whether reduced adult brain 5-HT synthesis affects body weight, food and water consumption in mice. There was no difference in body weight (F_(2,61)_ = 0.3954, *p* = 0.6751; Fig. 3c). However, experimental groups showed a statistical tendency towards quantity of water consumed (F_(2,61)_ = 3.059, *p* = 0.0542; Fig. S3b), with *Tph2*^*Δfl*/−^ drinking more water. This is in line with *Tph2*^*Δfl*/−^ consuming a significant higher quantity of food (F_(2,61)_ = 18.98, *p* < 0.0001; Fig. S3a) than *Tph2*^*Δ*+/+^ (*p* < 0.0001).

### Anxiety- and depression-like behavior in MS-exposed mice

**Figure 4:**
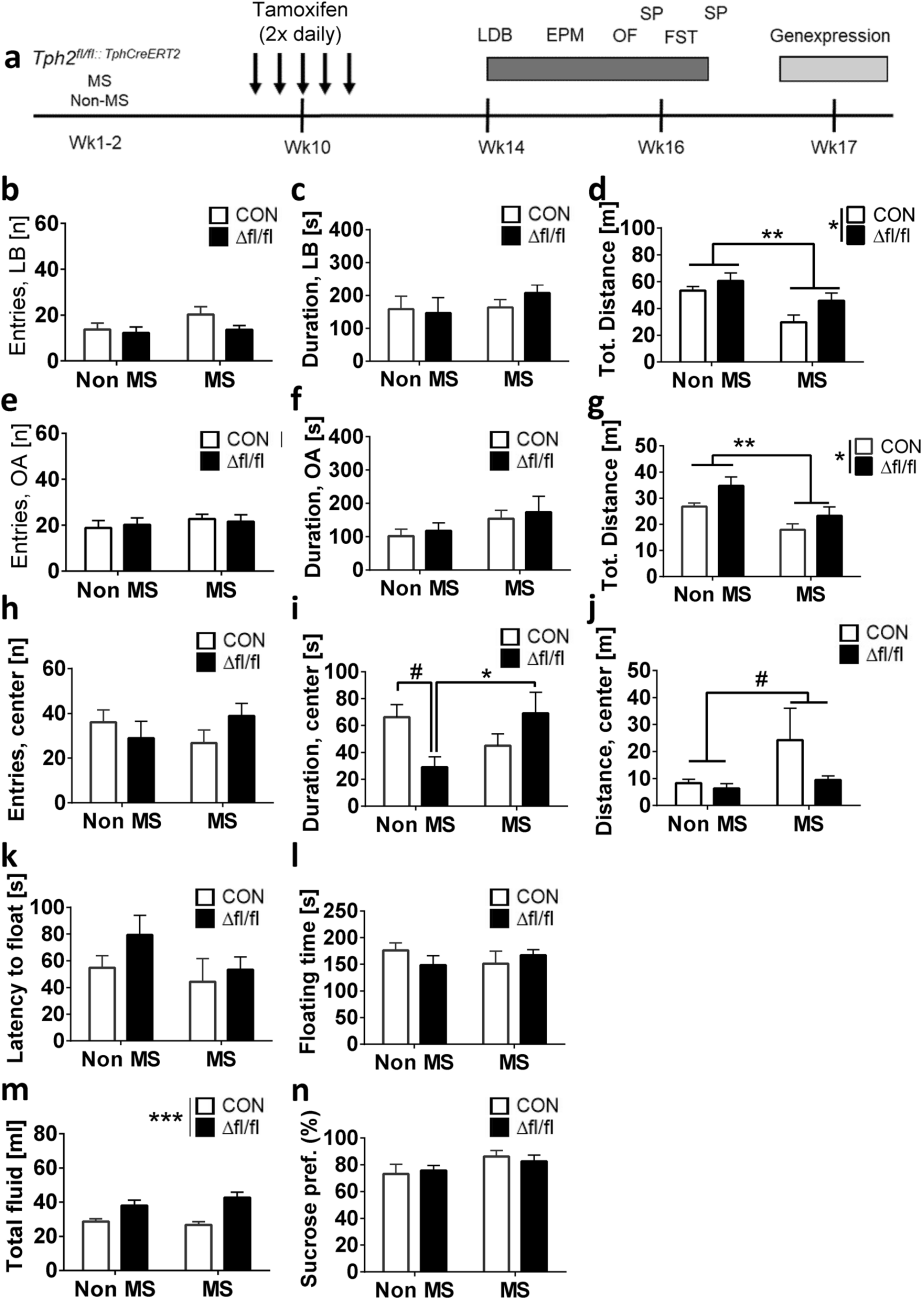
Anxiety-like, depression-like and exploratory behavior in MS-exposed *Tph2* icKO mice with homozygous genetic predisposition (*Tph2*^Δ*fl*/*fl*^). (**a**) Timelines for behavior testing; (**b**)–(**d**) LDB: light box visits, time in light box, total distance; (**e**)–(**g**) EPM: open arms visits, time in open arms and total distance; (**h**)–(**j**) OFT: center visits, time in center, total distance; (**k**),(**l**) FST: latency to float and floating duration; (**m**),(**n**) SPT: total fluid consumed and sucrose preference. Data are shown as mean ± SEM. #0.05 < *p* < 0.1, **p < 0.01 and ***p < 0.001.

Several studies suggested that the functionality of 5-HT in adulthood may be primed by early-life adversity to render an individual susceptible to emotion related disorders^38–40^. Therefore, Tg^Tph2creERT2^/*Tph2*^*fl*/*fl*^ mice, were subjected to MS stress from P2-P16, injected with TAM in adulthood and tested for anxiety- and depression-like behavior (Fig. 4a).

In the OF test, no MS × treatment interaction in the number of visits to the center (F_(1,32)_ = 2.275; *p* = 0.1413; Fig. 4h) was detected. Considering the total time spent in the center, a significant MS × treatment interaction effect (F_(1,32)_ = 6.562; *p* = 0.015; Fig. 4i) was observed. In the Non-MS group, *Tph2*^Δ*fl*/*fl*^ mice showed a tendency towards  less time in the center (*p* = 0.077) compared with *Tph2* CON. Inter-group comparison revealed that Non-MS *Tph2*^Δ*fl*/*fl*^ mice were more anxious than MS-exposed *Tph2*^Δ*fl*/*fl*^ mice (*p* = 0.035). Furthermore, MS exposure potentially increased total distance travelled in the aversive center (F_(1,32)_ = 6.562; *p* = 0.09; Fig. 4j).

In the LDT, mice were assessed based on their activities in the open compartment of the box. Two-way ANOVA revealed no significant MS × treatment interaction in measures of anxiety (F_(1,32)_ = 0.9622; *p* = 0.334, Fig. 4b,c) and overall distance (F_(1,32)_ = 0.6689; *p* = 0.4195) travelled. However, Non-MS mice covered longer distance than MS exposed mice (F_(1,32)_ = 12.49; *p* = 0.0013) and *Tph2*^Δ*fl*/*fl*^ mice were more active compared to *Tph2* CON mice (F_(1,32)_ = 4.672; Fig. 4d).

In the EPM, no significant MS × treatment interaction effect of was observed in visits to open arms (F_(1,31)_ = 0.1966; *p* = 0.6605; Fig. 4e), total time spent on open arms (F_(1,31)_ = 0.002; *p* = 0.9644; Fig. 4f) and the overall distance covered (F_(1,31)_ = 0.2576; *p* = 0.6153). However, MS exposed mice (F_(1,31)_ = 12.41; *p* = 0.0014) covered less distance than Non-MS mice, while *Tph2*^Δ*fl*/*fl*^ mice (F_(1,31)_ = 5.392; *p* = 0.027; Fig. 4g) covered longer distance than *Tph2* CON mice, independent of MS exposure.

Evaluation of depression-like behavior in the PST in both MS- and Non MS-exposed mice revealed no behavioral deficits (F_(1,31)_ = 0.3670; *p* = 0.549; Fig. 4k,l).

Furthermore the SPT showed no significant effects on sucrose preference (F_(1,31)_ = 0.3531; *p* = 0.5566; Fig. 4m). With respect to total fluid consumed, a significant main effect of treatment occurred (F_(1,31)_ = 20.9; *p* < 0.0001; Fig. 4n). *Tph2*^Δ*fl*/*fl*^ mice consumed more fluid than *Tph2* CON mice independent of aversive early-life stress experience. These outcomes indicate that reduction in adult brain 5-HT concentrations may not predispose to lack of pleasure that characterizes anhedonia but rather increases energy metabolism similar to constitutive *Tph2* KOmice^20,71^.

### Gene expression

**Figure 5:**
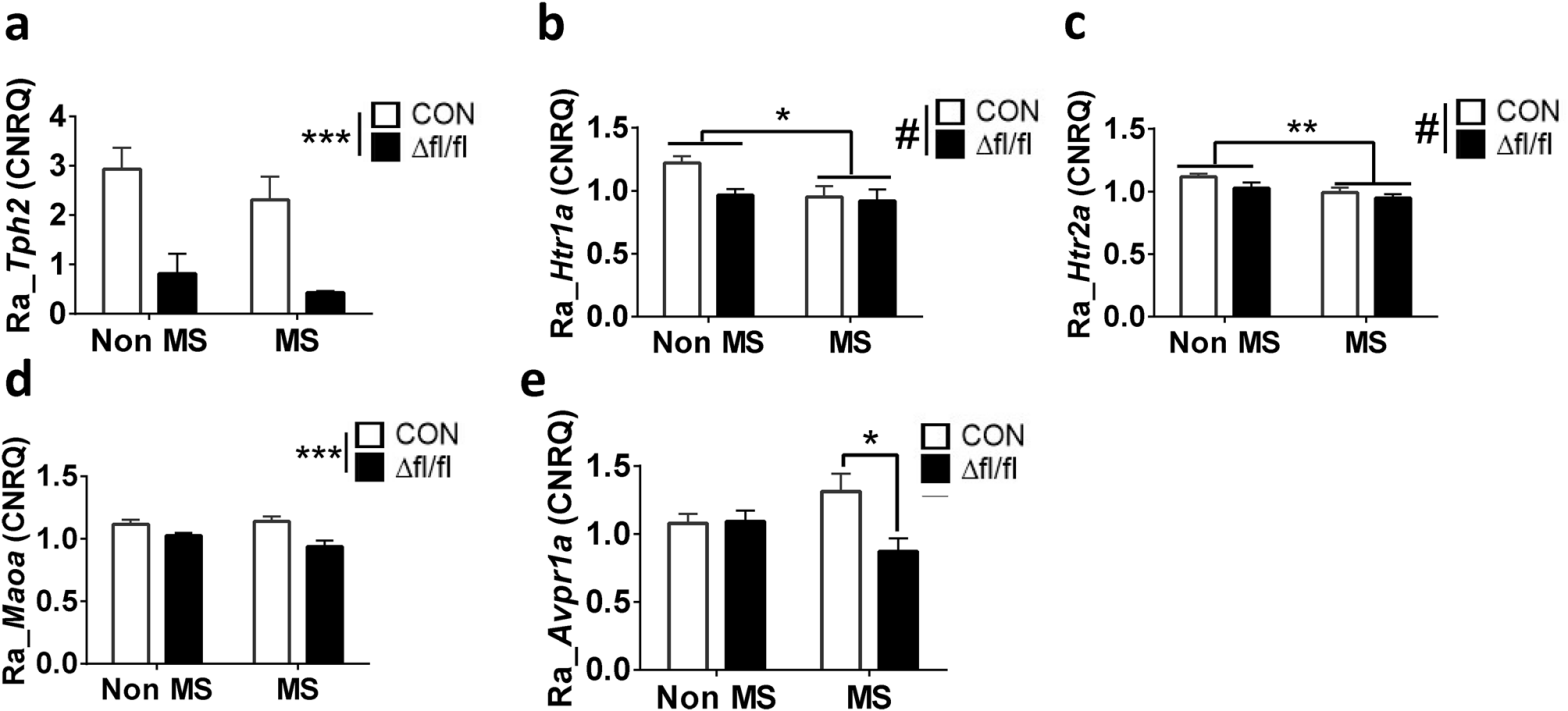
Expression of genes representing markers of 5-HT system function in the raphe region of MS-exposed *Tph2* icKO mice with homozygous genetic predisposition. After behavioral testing maternally separated (MS) and normally reared non-MS Tph2^fl/fl::Tph2CreERT2^ (fl/fl) mice, which were either injected at 10–12 weeks of age with TAM (Δfl/fl) or vehicle (CON) were analysed for differential expression of (**a**) *Tph2*, (**b**) *Htr1a*, (**c**) *Htr2a*, (**d**) *Maoa*, (**e**) *Avpr1*. Data are shown as mean ± SEM. #0.05 < *p* < 0.1, **p < 0.01 and ***p < 0.001.

The effect of MS-induced anxiety-related behavior on expression of genes, that are viewed as indicators of 5-HT system functionality in the raphe region, hippocampus and amygdala was also examined. *Tph2* expression indicated no significant MS × treatment interaction in the raphe region (F_(1,31)_ = 0.1104; *p* = 0.7419). However, a significant main effect of treatment (F_(1,31)_ = 30.05; *p* < 0.0001) was apparent (Fig. 5a). Thus, expression of *Tph2* was significantly reduced both in Non-MS and MS-exposed *Tph2icKO* mice which confirms the efficiency of conditional *Tph2* inactivation.

The relative expression of *Htr1a* in the raphe showed a significant main effect of treatment (F_(1,31)_ = 4.345; *p* = 0.0456; Fig. 5b) and a strong trend in the effect of MS (F_(1,31)_ = 3.597; *p* = 0.0672; Fig. 5d). No change in hippocampus and amygdala was detected (Fig. S4c).

Relative expression of *Htr2a* in the raphe region indicated no significant MS × treatment interactions (F_(1,31)_ = 0.3917; *p* = 0.536) mice but a significant main effect of MS (F_(1,31)_ = 7.599; *p* = 0.0097) and a trend towards treatment (F_(1,31)_ = 3.328; *p* = 0.0778; Fig. 5c). No alterations in hippocampus and amygdala were detected (Fig. S4b).

Assessment of *Maoa* expression responsible for the degradation of 5-HT in the raphe revealed a general effect of TAM treatment with significantly lower expression in *Tph2* icKO than *Tph2* CON mice (F_(1, 31)_ = 13.39; *p* = 0.0009; Fig. 5d). By contrast, a main effect of MS occurred in target brain areas, such as hippocampus (F_(1, 31)_ = 4.399; *p* = 0.0442; Fig. S4c, upper panel) and amygdala (F_(1, 30)_ = 3.068; *p* = 0.09; Fig. S4c, lower panel). *Avpr1a* was reported to influence anxiety in humans and rodents^34^. In this study a significant MS × treatment interaction (F_(1, 31)_ = 5.44; *p* = 0.0263; Fig. 5e) was observed in *Avpr1a* expression in raphe. *Post-hoc* analysis revealed *Avpr1a* expression in MS exposed *Tph2* CON mice was higher than MS-exposed *Tph2* icKO (*p* = 0.017). No differences were detected in the Non-MS cohort in both hippocampus and amygdala (Fig. S4d).

## Discussion

The first part of this study capitalized on the TAM induced Cre-mediated inactivation of *Tph2* to significantly reduce 5-HT synthesis in *adult* mouse brain. A transgenic *Tph2*-CreERT2 mouse line and the induction protocol described by (Weber et al. 2011) was used to induce a *Tph2* knockout in adulthood. Although few 5-HT positive cells in the raphe region remained, which may point to ineffective nuclear translocation of *Tph2*-CreERT2 probably due to reduced expression of *Tph2* in certain 5-HT immunoreactive neurons, we achieved similar recombination efficiencies as reported for other induced *Tph2* KO mice using *CMV*-CreERT2 transgenics as Cre driver line^41^ or using AAV-Cre viral injection into the rostral raphe nuclei^42^. Furthermore, measurement of 5-HT concentrations four weeks after TAM induction in the raphe, hippocampus and amygdala revealed efficiently reduced 5-HT concentrations in *Tph2* icKO comparable to constitutive *Tph2*^−/−^ null mutant mice^19^.

Efficient reduction of brain 5-HT and 5-HIAA within 4 weeks of TAM treatment has previously been reported^43^, which indicates that the TAM-mediated induction approach requires a relatively long time period before a significant deficiency in brain 5-HT concentration is established. However, the HPLC results showing low concentrations of 5-HT in *Tph2*^Δ+/+^ combined with highly increased 5HIAA levels at week 2 differed to previous results of non-injected *Tph2*^+/+^ mice in previous studies^19^. The increased 5-HT turnover in wildtype controls due to the stressful TAM injection protocol may represent a short time adaptive mechanism of the 5-HT system probably similar to restraint stress models^44^. Although, 5-HT concentrations in *Tph2*^*Δfl*/−^ and *Tph2*^Δ*fl*/*fl*^ mice did not differ in comparison with Tph2^Δ+/+^, the low 5-HIAA levels in both *Tph2*^*Δfl*/−^ and *Tph2*^Δ*fl*/*fl*^ groups reflect reduced 5-HT turnover similar to Tph2^−/−^ null mutant mice in the DRN^19^. Nevertheless, it seems that it takes at least four weeks in *Tph2*^*Δ*+*l*+^ mice to recover from the injection stress indicated by increasing 5-HT concentrations in the DRN similar to that of *Tph2*^+*l*+^ wildtype mice without any injection^19^. Interestingly, in the hippocampus and amygdala this effect on Tph2^Δ+/+^ is detected as well but it seems that it takes longer after TAM injections that 5-HT levels become degraded as compared to the raphe nuclei, which might be due to differentially edited or spliced *Tph2* mRNA isoforms with enhanced stability itself and or its coding proteins^45^ within serotonergic projections targeting these regions. Still, remaining 5-HT in induced *Tph2*^*Δfl*/−^ and *Tph2*^Δ*fl*/*fl*^ mice may arise from blood platelet 5-HT due to inefficient perfusion of the brain and/or from blood-borne 5-HTP derived from Tph1 enzymatic activity, which may cross the blood brain barrier and be converted into 5-HT by AADC^16,19^.

In line with the above explanation is an early increased concentration of NE in the brain coupled with expected reduction in 5-HT concentrations, which was probably caused by the injection procedure^44^. This shows that acute stress coupled with reduced 5-HT metabolism has an immediate influence on the NE and dopamine systems^42^. An effect, which normalized in the 4th week after TAM injections. Thus, the observed alterations in brain DA and NE concentrations add on the results from other studies^42,43^.

Interestingly, unlike *Tph2*^−/−^ mice, which exhibit reduced anxiety-like behavior^21,22,46^, *Tph2*^*Δfl*/−^ and *Tph2*^*Δfl*/*fl*^ mice showed no differences in anxiety- and depression-like behavior. However, locomotor hyperactivity or panic-like responses in aversive inescapable novel environment in *Tph2*^−/−^ mice^32^ and locomotor hyperactivity as described for *Tph2*^*icko*^ mice^42^ were specifically observed in *Tph2*^*Δfl*/−^, which resemble genetic heterozygous knockout mice with a 20–30% reduction in raphe 5-HT levels^19^. This is in line with reports of involvement of DR and MnR 5-HT in responses to future threat^47,48^. In contrast, *Tph2*^*Δfl*/*fl*^ did not differ in any behavioral assessment compared to *Tph2*^Δ+/+^mice. Indeed, it points towards a two-hit effect^49^. Based upon a primed heterozygous genetic background during development, an induced or acquired 5-HT deficiency may provoke more pronounced panic-like flight responses in inescapable aversive conditions, while a development without changes in the 5-HT system like in *Tph2*^Δ*fl*/*fl*^ is able to compensate further environmental or genetic impacts in adulthood despite the near complete ablation of 5-HT synthesis.

### Interaction of early life MS stress and aberrant adult brain 5-HT on behavior

In order to further investigate this effect during early development, we used early life MS, which has been commonly used to study G × E interaction on anxiety and depression in mice^50,51^ and has been shown to impact anxiety-like behaviors in rodents via the 5-HT system^52,53^. Exposure of wildtype mice, on a mixed c57BL6/J–129S6/Sv background, to MS significantly decreased distance travelled and time spent in the center of the OFT, while MS had no effect in *Tph2* KI mice^54^. Here, we could show that MS produced a similar effect in vehicle injected *Tph2*^*fl*/*fl*^ control mice and this effect was rescued by inducing a Tph2 deficiency. This shows that MS exposure and reduced brain 5-HT differentially influence anxiety-like behavior and may compensate each other.

Thus, this study complements numerous studies, which focused specifically on manipulation of *Tph2* expression in adult mouse brain and its effect on emotion-related behavior^42^. Aberrant 5-HT neurotransmission in the adult brain either via pharmacological interventions^55,56^ or induced gene inactivation^42,43^ was unable to destabilize behavioral adaptive mechanisms that are established during early brain maturation.

Notably, neither MS nor induced adult 5-HT deficiency altered preferences for sucrose solution indicating no differences in hedonic-like behaviors. Furthermore, no alterations in depression-like behavior in the PST were observed in this study. One explanation may be that maternal care on reunion with pups increased and this may have dissolved the MS effect on depression-like behaviors. Thus, it may well be that the MS protocol used was not robust enough to impact depression-like behavior in the mice^57^ and requires further studies including investigation of maternal behaviour. MS exposure neither affected hedonic behavior in rats^58^, nor did it impact anxiety- and depression-related behavior in C57BL/6 mice^59^. Even in *TPH2* KI mice, which have reduced brain 5-HT concentrations throughout life, MS exposure did not alter anxiety- and depressive-like behaviors^60^. By contrast, some studies have associated MS exposure with abnormal behavior and stress induced alteration in neurotransmitter concentration^61,62^. Noteworthy, most rodent studies, which found an association between MS with behavioral alterations, were largely done in rats^62–64^. Indeed, some C57BL/6 mouse strains appear to be resilient to neonatal MS stress^57,59,65–70^, which may explain the weak MS effects observed in this study.

However, *Tph2* icKO mice consumed more fluid than *Tph2* CON mice, which points towards an acute metabolic effect of 5-HT deficiency in *Tph2* KO mice^71^. The observed increase in food, water and percentage sucrose consumption by *Tph2*^*Δfl*/−^ and *Tph2*^Δ*fl*/*fl*^ mice has also been reported in *Tph2*^−*l*−^ mice^20,71^, which reflects an increased energy need, rather than altered anhedonia as a symptom of depressive-like behavior. This supports the assertion that strong reductions in 5-HT metabolism in adulthood are implicated in the pathophysiology of eating disorders through various hormonal and receptor systems^72,73^, independent of 5-HT functions during development.

### Gene expression in raphe, hippocampus and amygdala

Early-life stress does not alter the expression of *Tph2* in mice^60^ and rats^52^. In contrast, an association between MS exposure and raphe region-specific reduction in *Tph2* expression of C56BL/6 J mice has been reported^74^. Here, the relative expression of *Tph2* in *Tph2* icKO was significantly lower than *Tph2* CON mice in raphe, while MS exposure alone did not alter *Tph2* expression.

We detected no differences in expression of *Htr1a*, *Htr2a and Avpr1a* in target regions of MS-naive mice. This confirms our earlier work, which also reporting no change in expression of *Htr1a and Htr2a* genes in non-stressed *Tph2* conditional KO mice^75^. A slightly altered expression of *Maoa* in amygdala and hippocampus may point to specific compensatory mechanisms due to MS^53^. Furthermore, MS exposed mice showed altered expression of *Htr1a* and *Htr2a* in the raphe independent of Tph2 icKO, which is in line with previous MS studies^76,77^. Alterations in 5-HT receptors in target regions of 5-HT neurons have previously been associated with altered anxiety and exploratory behavior^78^. Here, this may explain the dampening effect of MS on total locomotor activity in OF and EPM.

However, a *Tph2* icKO reduced expression of *Maoa* in the raphe region, pointing towards a direct effect of strong 5-HT depletion on MaoA dependent 5-HT turnover processes. Thus, effects mediated by adverse life experience and associated with altered *Maoa* expression^34^ may be prohibited in *Tph2* icKO mice through a dysfunctional 5-HT system in adulthood. Interestingly, *Avpr1a* expression was only affected in the raphe after the mice were maternally separated. We did not find altered *Avpr1a* expression in the hippocampus, which was correlated after late adverse life experiences with reduced anxiety-like behavior^34^. Nevertheless, Avpr1a is present in the dorsal raphe, the mesencephalic central gray and the caudal linear raphe^79^. Additionally, an *Avpr1a* knockout as well as pharmacological blockade of *Avpr1a* function in rodents reduced aggression and resulted in anxiolytic and anti-depressive-like effects^80,81^. Thus, the potential anxiolytic effect observed in MS *Tph2* icKO mice may be directly attributable to altered *Avpr1a* expression. This highlights the interaction of arginine vasopressin-dependent signaling with the 5-HT system in the brainstem following MS as a potential therapeutic target for treating emotional dysregulation.

In conclusion. Our findings establish a "double-hit" experimental model to study the behavioral and neurobiological consequences of 5-HT deficiency in adulthood in interaction with early-life stress experience potentially affecting emotion regulation and the pathogenesis of depressive disorders.

## Supplementary Information

Supplementary Information.
