# Supplementary material for: Serotonin deficiency induced after brain maturation rescues consequences of early life adversity
Source: Sci Rep. 2021 Mar 8;11:5368. doi: 10.1038/s41598-021-83592-4 (PMC7940624; doi:10.1038/s41598-021-83592-4)
Supplement: Supplementary file 1 — Supplementary Information. [file 41598_2021_83592_MOESM1_ESM.doc]

**Serotonin deficiency induced after brain maturation rescues consequences of early life adversity**

B Aboagye^1,7^, T Weber^4,5^, HL Merdian^6^, D Bartsch^4^, KP Lesch^1,2,3^*, J Waider^1^*

^1^Division of Molecular Psychiatry, Laboratory of Translational Neuroscience, Center of Mental Health, University of Würzburg, Germany

^2^Laboratory of Psychiatric Neurobiology, Institute of Molecular Medicine, I.M. Sechenov First Moscow State Medical University, Moscow, Russia

^3^Department of Psychiatry and Psychology, School for Mental Health and Neuroscience (MHeNS), Maastricht University, Maastricht, The Netherlands

^4^Department of Molecular Biology, Central Institute of Mental Health, Medical Faculty Mannheim/Heidelberg University, Mannheim, Germany

^5^MEDIAN Clinic Wilhelmsheim, Oppenweiler, Germany

^6^School of Psychology, University of Lincoln, Lincoln, UK

^7^Department of Biomedical and Forensic Science, School of Biological sciences, University of Cape Coast, Cape Coast, Ghana

Supplementary Results


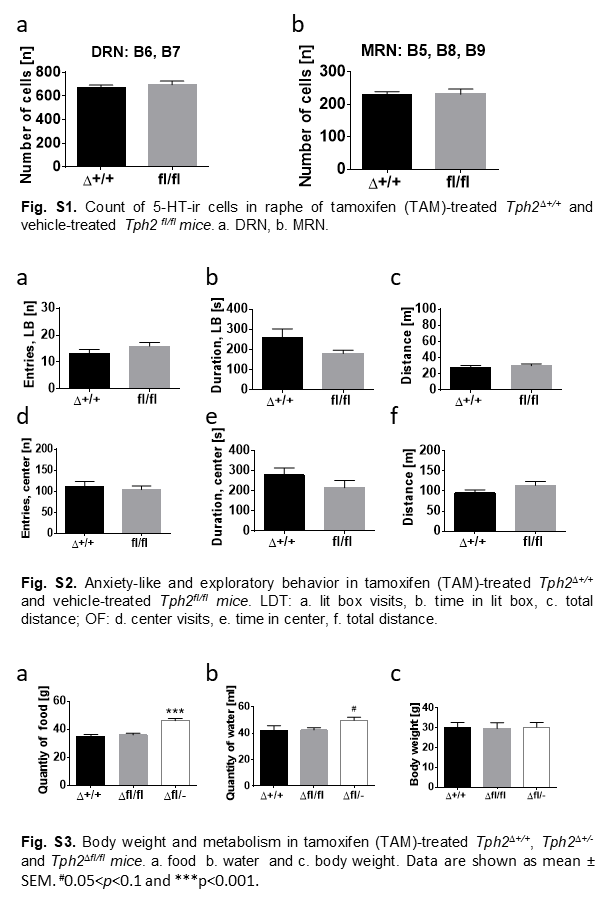


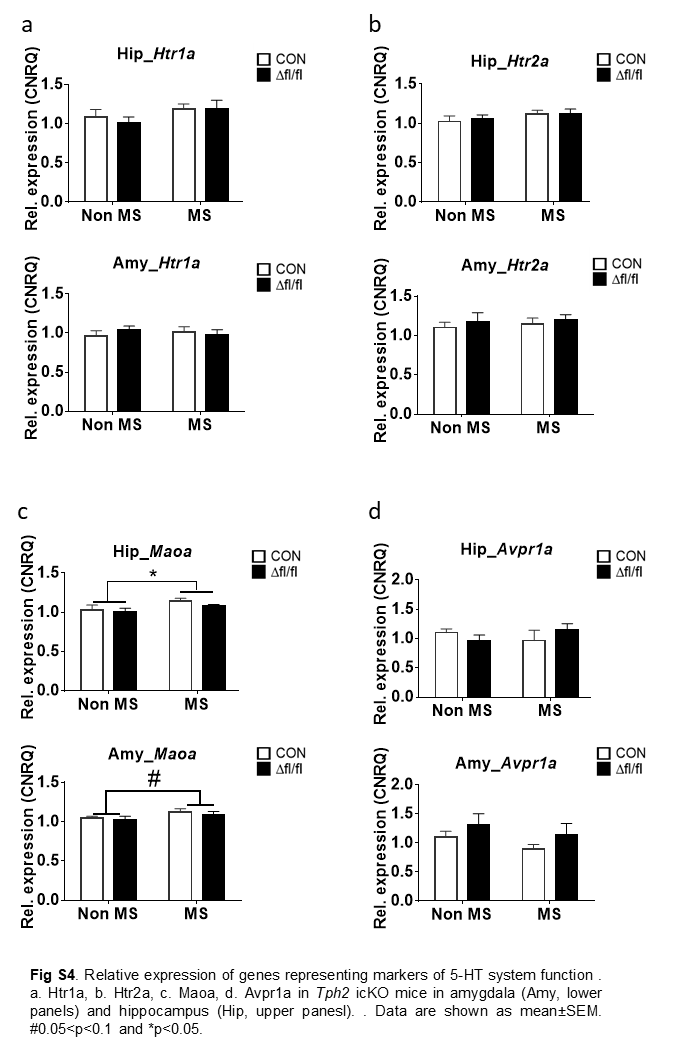


Supplementary Methods

Light-dark transition test. The LDT consisted of a transparent Perspex ‘light’ compartment (40x40x27cm) and a black opaque Perspex ‘dark’ compartment (40x20x27cm). Mice were placed into the dark compartment and latency to enter the lit compartment, the distance traveled, and the time spent in the lit compartment were assessed. The dark chamber contained a small opening at floor level (5x5cm) and was covered by a removable lid. The illumination in the dark compartment was between 0 and 10 lx, whereas illumination in the light compartment was 100 lx. Once mice were placed in the dark section of the box, their behavior was automatically recorded for 10 min.

Open-field test. The OF consisted of a black quadratic box (50x50x40 cm), semi-permeable to infrared light (Post et al., 2011). Illumination at floor level was between 50 and 100 lx (from the walls to the center of the arena). The arena was divided into a 30x30 cm center zone and the surrounding periphery. Mice were individually placed in one corner of the arena and automatically recorded for 30 min. The distance traveled, and time spent moving were used to evaluate locomotor activity; frequency of entry into the center and the time spent in the center of the arena were considered as a measure of anxiety-like behavior.

Elevated-plus maze test. The EPM was made of grey Perspex, raised to about 60 cm high above the floor. The apparatus comprises two open arms (30x5x0.25 cm) and two closed arms (30x5x15 cm) which intersect at a central platform (5x5 cm). Illumination intensity was 200 lx on the open arms, 120 lx on the central platform and 40 lx on the closed arms. Mice were positioned at the center, facing one of the open arms, and allowed to freely explore the maze for 10 min. The number of arm entries, the time spent in the open and closed arms and the total distance traveled were recorded using an automated video tracking system.

Porsolt swim test (PST). Mice were introduced to a transparent cylinder (height 20 cm, diameter 15 cm) filled with water (25°C, height 12 cm) for 5 min. With the critical variable being immobility time, defined as the absence of directed movements of animals’ head and body as well as the latency to immobility, mice were constantly tracked. Short periods of slight activity, necessary to maintain their heads above water, were included within the time spent immobile.

Sucrose preference test (SPT). Here, mice choose between two bottles, one with 2.5% sucrose solution and the other with tap water. Baseline sucrose and water consumption (calculated by weighing the bottles every 24h) was measured for 6 days in the fifth week after TAM injection. The position of the bottles in the cage was switched every day. Sucrose preference was calculated as a percentage of the consumed sucrose solution from the total amount of liquid consumed using the following formula: Sucrose Preference = (V (sucrose solution) / V (sucrose solution) + V (water)) x 100%.
